# Trends in injury-related mortality among residents of Jiangsu Province from 2012 to 2021: an age-period-cohort analysis

**DOI:** 10.3389/fpubh.2024.1373238

**Published:** 2024-06-11

**Authors:** Wencong Du, Rong Wang, Xikang Fan, Xun Wu, Jie Yang, Jinyi Zhou, Hao Yu

**Affiliations:** ^1^Department of Noncommunicable Chronic Disease Control, Jiangsu Provincial Center for Disease Control and Prevention, Nanjing, China; ^2^Department of Epidemiology and Biostatistics, School of Public Health, Southeast University, Nanjing, China; ^3^Department of Child and Adolescent Health Promotion, Jiangsu Provincial Center for Disease Control and Prevention, Nanjing, China

**Keywords:** epidemiology, injury, age-period-cohort analysis, mortality, trend

## Abstract

**Objective:**

We investigated the temporal trends and examined age-, period-, and cohort-specific effects of injury-related deaths among residents in Jiangsu to provide evidence for future injury prevention.

**Methods:**

This study included 406,936 injury deaths from the Jiangsu provincial population death registration system. The average annual percent change (AAPC) in age-standardized mortality rates (ASMRs) was analyzed using joinpoint regression. Age-period-cohort models were generated to explore the effects of age, period, and birth cohort effects on mortality risk.

**Results:**

ASMRs for all injuries (AAPC = −2.3%), road traffic accidents (AAPC = −5.3%), suicide (AAPC = −3.8%), and drowning (AAPC = −3.9%) showed a downward trend during 2012–2021(all *p* < 0.05), while unintentional falls showed an upward trend (AAPC = 5.1%, *p* < 0.05). From 2012 to 2021, the age-standardized mortality rates (ASMRs) for four primary types of injuries consistently exhibited higher among males compared to females, with rural regions displaying higher ASMRs than urban areas. Trends in ASMRs for road traffic accidents, drowning, and unintentional falls by sex and urban/rural areas were consistent with overall trends. Significant age, cohort, and period effects were identified in the trends of injury-related deaths for both sexes in Jiangsu. The age effect showed that the highest age effect for injury-related deaths was for the ages of 85 years and above, except for suicide, which was for the ages 80–84 years. Between 2012 and 2021, the period effect on road traffic accidents declined, while that on accidental falls increased. Initially, the period effect on suicide decreased but then rose, peaking in 2012 with a Relative Risk (RR) of 1.11 (95% *CI*: 1.04–1.19). Similarly, the period effect on drowning initially declined before rising, with the highest effect observed in 2013, at an RR of 1.12 (95% *CI*: 1.07–1.19). The highest cohort effects for road traffic accidents were observed in the 1957–1961 group, for accidental falls in the 1952–1956 group, and for both drowning and suicide in the 1927–1931 group.

**Conclusion:**

The mortality rate of unintentional falls has been increasing. Older adults are at high risk for the four leading injuries. The improvements in mortality rates can be attributed to advancements in education, urbanization, and the promulgation and implementation of laws and policies.

## 1 Introduction

Injuries represent a significant global public health concern, due to their prevalence and high rates of disability and mortality. They seriously affect the health and quality of life of individuals causing substantial burdens for both society and families (1, 2). According to the World Health Organization (WHO) estimated, approximately 4 million people die from injuries worldwide every year (3). In China this figure is particularly striking, with an estimated 700,000–800,000 fatalities from various injuries annually (4). With rapid social development, injuries have become the fifth leading cause of death, following malignant tumors, cerebrovascular diseases, respiratory diseases, and heart disease (5). Various types of injuries have distinct characteristics. Despite a noticeable reduction in recent decades (6, 7), road traffic injuries continue to be the leading cause of both death and premature mortality on a global scale. In low- and middle-income countries, unintentional fall-related deaths account for more than 80%, and over 90% of drowning deaths occur. In China, the most populous country in the world, suicide is the leading cause of death among people aged 15–34 years and ranks as the fifth leading cause of death in the general population (8).

Over the past few decades, China has experienced profound social transformations in its economy, demography, health, and politics, which have influenced mortality rates associated with major types of injuries (9, 10). The study findings suggest that age, period, and cohort effects may all play a role in injury-related mortality, with variations observed across countries likely attributable to differences in racial, sociocultural, educational, and legal factors (11–13). Jiangsu Province, located on the east coast of China, has experienced robust economic development and significant social advancement, resulting in overall improvements in population health. However, Jiangsu faces challenges including an increased risk of falls among the older adult due to its aging population, transportation-related accidents due to the high vehicle traffic, injuries associated with manufacturing activities such as firearm and cutting incidents, and a heightened risk of drowning due to numerous water bodies within the province. While previous studies have examined the trend of injury-related mortality rates at the national level (14–16), evidence at the subnational level in mainland China remains insufficient. The understanding of injury-related mortality trends in Jiangsu Province, including variations across different age groups, periods, and populations, is not yet clear. Therefore, this study aimed to analyze the trends of four injury-related mortality rates—road traffic accidents, unintentional falls, suicide, and drowning—in Jiangsu’s population from 2012 to 2021 using joinpoint regression models. An age-period-cohort model was used to estimate the effects of age, period, and cohort on these four injury-related mortality rates to provide scientific information for future injury interventions.

## 2 Method

### 2.1 Study population

The study collected death data from 2012 to 2021 through the Jiangsu provincial population death registration system, which is maintained by the Jiangsu Province Centers for Disease Control and Prevention (CDC). Demographic details, including age and sex, were obtained from the public security household registration department of each municipality. Concurrently, the underlying causes of death, as documented in medical records or certified by relevant certificates, were ascertained by professionals.

Suqian city accounts for about 6% of Jiangsu’s population. From 2012 to 2014, the death data from Suqian city was excluded from the study due to non-compliance with the Jiangsu Provincial Data Quality Control Standards. Since 2014, the death surveillance data from all counties and districts of Jiangsu province have been qualified and included in the analysis.

### 2.2 Ascertainment of outcomes

The cause of death was classified according to the International Statistical Classification of Diseases and Related Health Problems, 10th Revision (ICD-10). We examined all injuries (V01-Y89) and four specific severe injuries with high mortality rates, namely, road traffic accidents (V01-V04, V06, V09-V80, V87, V89, V99), unintentional falls (W00-W19), drowning (W65-W74), and suicide (X60-X84, Y87.0) (17).

### 2.3 Statistical analysis

A joinpoint regression model was used to describe the continuous changes in the temporal trends of four types of injuries among residents in Jiangsu from 2012 to 2021, using joinpoint regression program software version 4.9.1.0 (National Cancer Institute). The basic principle of the joinpoint regression model is to describe trends by connecting several different line segments at “joinpoints” and identifying points where the linear slope of a trend changes in a statistically significant way over time (18). In order to assess the magnitude of the time trends for mortality rates, the annual percent change (APC) and average annual percent change (AAPC) were calculated (19). A two-tailed *p*-value of less than 0.05 was considered to indicate statistical significance.

The age-period-cohort model evaluates mortality risk within a population for a specific year, by examining both the cumulative impact of risk factors from birth and also allowing for the analysis of the independent effect of age, period, and cohort on temporal trends in injury mortality (20). The age-period-cohort model provides a useful parametric framework that complements standard nonparametric description methods. In this model, the collected data were stratified into consecutive 5-year age groups and successive 5-year intervals. Injury-related mortality rates were recorded for consecutive 5-year age groups, consecutive 1-year periods, and corresponding consecutive 5-year birth cohorts. Age-period-cohort analysis using the eigenvalue estimator method provided estimated coefficients for the impact of age, period, and cohort effects. These coefficients were converted to exponential values [exp(coef.) = e^coef.], indicating the relative risk (RR) of mortality for a particular age, period, or birth cohort relative to the average of all ages, periods, or birth cohorts combined. The analysis was implemented by the APCG1 package in R programming language.

## 3 Results

A total of 406,936 injury deaths were included in the study, comprising 134,794 deaths from road traffic accidents, 115,969 deaths from unintentional falls, 41,286 deaths from suicide, and 37,757 deaths from drowning. The ASMRs of injury-related deaths in Jiangsu province from 2012 to 2021 were shown in Table 1. Generally, there was a downward trend in the death rate from traffic accidents, suicide, and drowning (*t* = −8.9 to −2.5, AAPC = −5.3% to −3.8%, *p* < 0.05), while the rate of unintentional falls increased (*t* = 17.7, AAPC = 5.1%, *p* < 0.001).

**Table 1:** Leading injury mortality rate in Jiangsu from 2012 to 2021 (/10^5^).

Year	Total		Road traffic accident		Unintentional falls		Suicide		Drowning	
	CMR	ASMR	CMR	ASMR	CMR	ASMR	CMR	ASMR	CMR	ASMR
2012	49.72	35.69	24.12	18.63	9.15	5.33	7.54	5.06	6.84	5.44
2013	51.97	35.93	23.20	17.52	10.26	5.71	6.98	4.75	6.72	5.31
2014	45.88	30.18	20.27	15.09	11.62	6.02	6.44	4.24	5.39	4.00
2015	49.68	31.06	19.94	14.42	13.77	6.59	6.28	4.19	6.06	4.36
2016	53.53	31.17	19.24	13.13	15.62	6.96	6.26	4.03	5.98	4.27
2017	55.20	31.40	20.49	13.77	17.22	7.61	6.10	3.88	5.77	3.97
2018	54.92	30.28	19.79	12.70	18.37	7.84	5.99	3.90	5.78	4.01
2019	56.18	29.91	19.69	12.23	19.52	7.99	5.67	3.82	5.80	3.85
2020	58.31	30.09	19.70	11.77	20.12	8.24	5.38	3.67	6.19	4.29
2021	60.32	29.51	20.37	11.62	21.34	8.18	5.10	3.63	5.94	3.86
AMR	43.12	25.96	20.68	14.09	15.70	7.05	6.17	4.12	6.05	4.34
AAPC(%)	2.6	−2.3	−2.1	−5.3	9.9	5.1	−3.7	−3.8	−1.7	−3.9
*p*-value	0.001	<0.001	0.009	<0.001	<0.001	<0.001	<0.001	<0.001	0.084	0.014

Injury: includes injury, poisoning and other specific consequences of external causes; AAPC, average annual percentage change; AMR, average mortality rate; CMR, crude mortality rate; ASMR, age-standardized mortality rate.

We analyzed the changes in the ASMRs for the four categories by joinpoint regression, as shown in Table 2. Between 2012 and 2021, fatalities from suicide and drowning declined more rapidly among females, while those from road traffic accidents decreased more quickly among males. Additionally, the increase in fatalities from accidental falls was greater among females compared to males. Rural areas experienced a more significant decline in fatalities from road traffic accidents and drowning than urban areas. Conversely, the risk of accidental falls is increasing in urban areas. Suicide mortality is decreasing in rural regions but is on the rise in urban areas. Notably, 2014 marked the convergence point for changes in road traffic accident mortality rates across genders.

**Table 2:** Changes in the trends of leading injury-related deaths by sex and urban/rural area in Jiangsu Province from 2012 to 2021.

Type	Trend 1			Trend 2			AAPC(95% *CI*)	*p*-value
	Year	APC (95% *CI*)	*p*-value	Year	APC (95% *CI*)	*p*-value		
**Male**								
Road traffic accident	2012–2014	−10.6 (−19.0 ~ −1.4)	0.033	2014–2021	−4.3 (−5.5 ~ −3.0)	<0.001	−5.7 (−7.5 ~ −4.0)	<0.001
Unintentional falls	2012–2018	5.7 (2.2 ~ 9.3)	0.008	2018–2021	2.5 (−7.2 ~ 13.3)	0.547	4.6 (1.5 ~ 7.9)	0.004
Suicide	2012–2014	−6.1 (−12.0 ~ 0.2)	0.054	2014–2021	0.7 (−0.2 ~ 1.6)	0.090	−0.8 (−2.0 ~ 0.4)	0.169
Drowning	2012–2021	−12.7 (−26.8 ~ 4.1)	0.104	2014–2021	−0.6 (−2.9 ~ 1.8)	0.560	−3.4 (−6.5 ~ −0.2)	0.039
**Female**								
Road traffic accident	2012–2014	−10.8 (−20.3 ~ −0.1)	0.049	2014–2021	−1.9 (−3.4 ~ −0.5)	0.020	−4.0 (−6.0 ~ −1.9)	<0.001
Unintentional falls	2012–2017	9.2 (6.2 ~ 12.3)	<0.001	2017–2021	5.5 (1.5 ~ 9.8)	0.017	7.6 (5.7 ~ 9.5)	<0.001
Suicide	2012–2014	−10.0 (−17.5 ~ −1.8)	0.027	2014–2021	−1.3 (−2.5 ~ −0.2)	0.032	−3.3 (−4.9 ~ −1.7)	<0.001
Drowning	2012–2014	−13.4 (−28.1 ~ 4.2)	0.102	2014–2021	−0.9 (−3.3 ~ 1.6)	0.405	−3.8 (−7.1 ~ −0.4)	0.028
**Urban**								
Road traffic accident	2012–2014	−7.4 (−15.9 ~ 2.0)	0.098	2014–2021	−2.6 (−3.9 ~ −1.3)	0.003	−3.7 (−5.4 ~ −1.9)	<0.001
Unintentional falls	2012–2014	−0.5 (−4.1 ~ 3.2)	0.735	2014–2021	8.1 (7.6 ~ 8.6)	<0.001	6.1 (5.4 ~ 6.9)	<0.001
Suicide	2012–2014	−2.6 (−11.5 ~ 7.3)	0.520	2014–2021	3.6 (2.2 ~ 4.9)	0.001	2.2 (0.3 ~ 4.0)	0.020
Drowning	2012–2014	−8.6 (−20.8 ~ 5.4)	0.165	2014–2021	0.8 (−1.1 ~ 2.8)	0.311	−1.3 (−4.0 ~ 1.3)	0.320
**Rural**								
Road traffic accident	2012–2015	−9.1 (−14.1 ~ −3.8)	0.008	2015–2021	−3.3 (−5.2 ~ −1.5)	0.006	−5.3 (−6.9 ~ −3.6)	<0.001
Unintentional falls	2012–2017	7.5 (6.6 ~ 8.4)	<0.001	2017–2021	2.3 (1.0 ~ 3.5)	0.005	5.1 (4.6 ~ 5.7)	<0.001
Suicide	2012–2014	−8.7 (−12.8 ~ −4.5)	0.004	2014–2021	−2.3 (−2.9 ~ −1.7)	<0.001	−3.8 (−4.6 ~ −3.0)	<0.001
Drowning	2012–2014	−13.3 (−27.0 ~ 2.9)	0.085	2014–2021	−1.1 (−3.3 ~ 1.2)	0.279	−3.9 (−7.0 ~ −0.8)	0.014

APC, annual percentage change; AAPC, average annual percentage change; CI, confidence interval.

**Figure 1:**
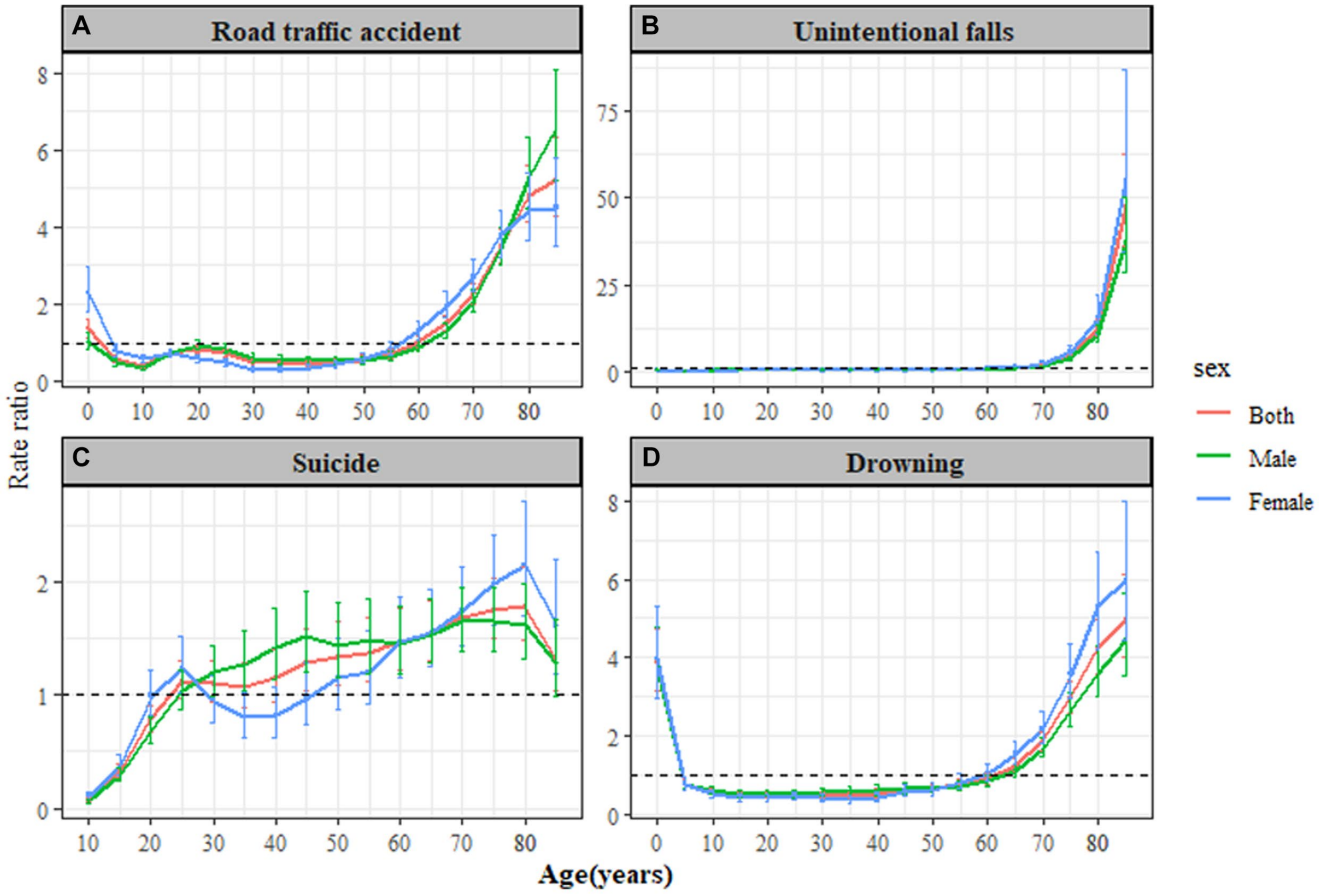
Longitudinal age curves of leading injury-related deaths rate by gender in Jiangsu Province, from 2012 to 2021. **(A)** Road traffic accident, **(B)** Unintentional falls, **(C)** Suicide, and **(D)** Drowning.

The age, period, and cohort effects on four types of injury-related deaths varied among the population of Jiangsu. As shown in Figure 1A, the greatest age effect for death from road traffic accidents was observed in those aged 85 years and above, with an effect coefficient of 1.65 and a RR of 5.21 (95% CI: 4.31–6.30). The trends in age-period-cohort (APC) effects on road traffic accident mortality by sex were generally consistent with those of the entire population. In Figure 1B, the highest age effect for death from unintentional falls was observed in individuals aged 85 years and above, with an effect coefficient of 3.86 and a RR of 47.67 (95% *CI*: 36.28–62.63). In Figure 1C, the most notable age effect for death from suicide was found in the 80–84 years age group, with an effect coefficient of 0.57 and a RR of 1.78 (95% *CI*: 1.48–2.13). In Figure 1D, the highest age effect for death from drowning was also observed in individuals aged 85 years and above, with an effect coefficient of 1.60 and a RR of 4.98 (95% *CI*, 4.04–6.13).

**Figure 2:**
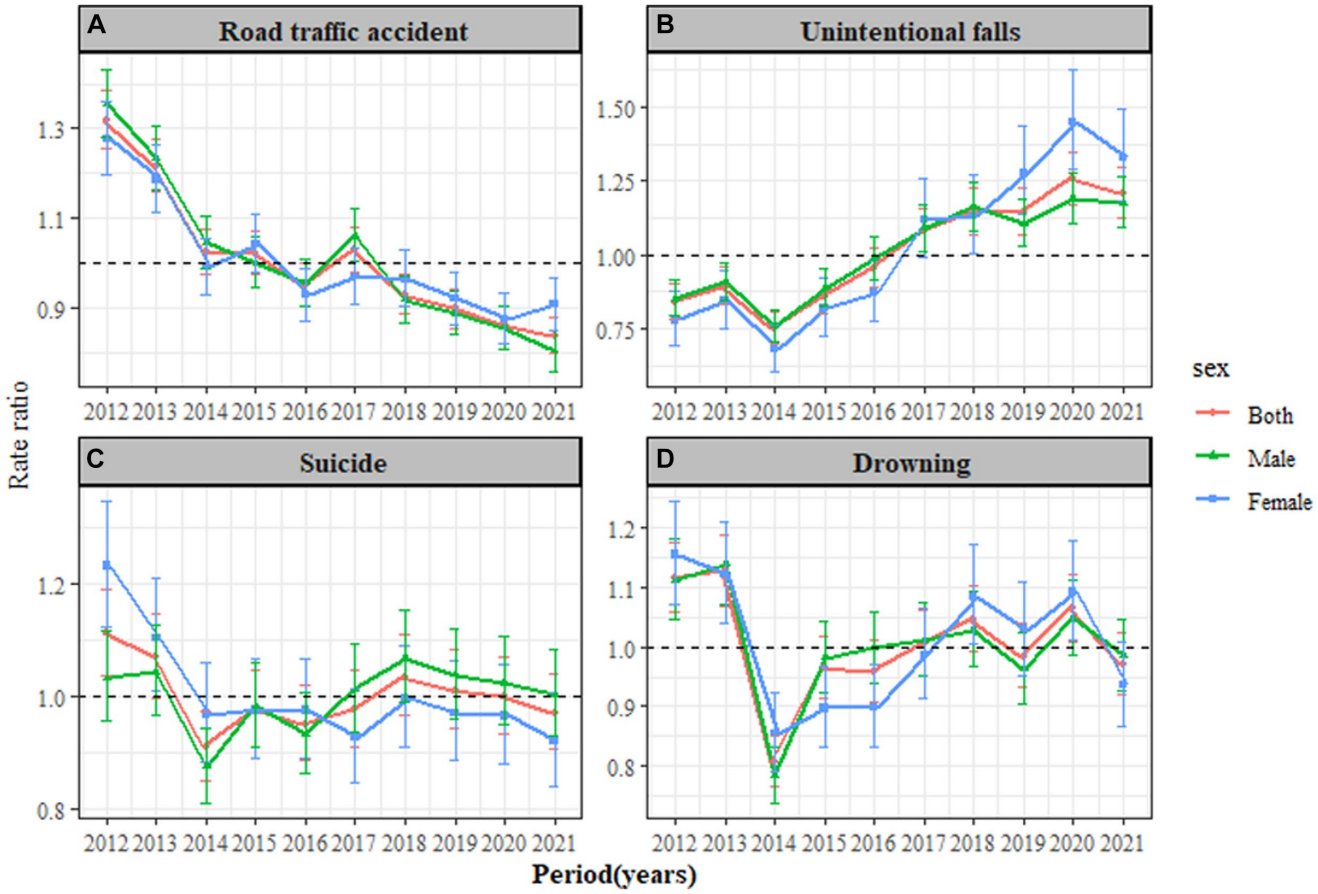
Period RRs of leading injury-related deaths rate by gender in Jiangsu Province, from 2012 to 2021. **(A)** Road traffic accident, **(B)** Unintentional falls, **(C)** Suicide, and **(D)** Drowning.

In Figure 2A, the period effect for road traffic accidents showed a gradual decrease, from an effect coefficient of 0.28 in 2012 to −0.17 in 2021. In Figure 2B, the period effect for unintentional falls indicated a steady increase, with the effect coefficient rising from −0.29 in 2014 to 0.23 in 2020. In Figure 2C, the period effect for suicide experienced a significant change in 2012, reaching the highest observed effect that year, with a relative risk (RR) of 1.11 (95% *CI*: 1.04–1.19). In Figure 2D, the peak period effect for drowning was observed in 2013, with an RR of 1.13 (95% *CI*: 1.07–1.19).

**Figure 3:**
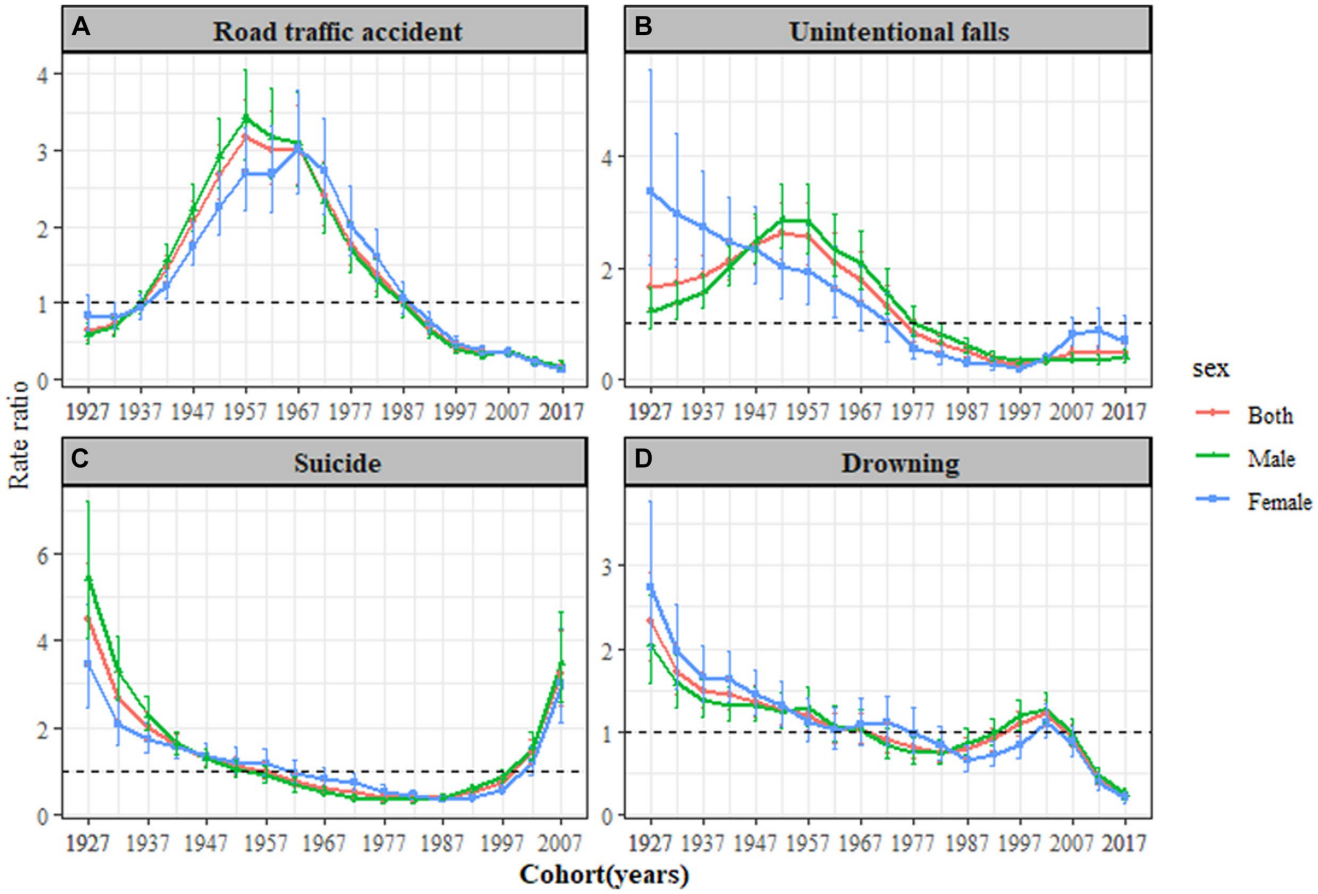
Cohort RRs of leading injury-related deaths rate by gender in Jiangsu Province, from 2012 to 2021. **(A)** Road traffic accident, **(B)** Unintentional falls, **(C)** Suicide, and **(D)** Drowning.

In Figure 3A, an increasing cohort effect for traffic accidents was observed among individuals born between 1927 and 1957, with the peak effect occurring around the cohorts born from 1957 to 1961, showing a RR of 3.17 (95% CI: 2.73–3.67) for the 1962 cohort. In Figure 3B, the mortality cohort effect for unintentional falls showed an initial increase followed by a subsequent decrease from the 1927 cohort onward, with the highest effect noted for the 1952–1956 cohort, having an RR of 2.63 (95% *CI*: 2.17–3.18). Notably, age effects were more pronounced in women aged 60 years and older and were relatively weaker in women compared to men. In Figure 3C, the mortality cohort effect for suicide showed a decrease followed by an increase from the 1927 cohort onward, with the highest cohort effect observed for the 1927–1931 cohort, which had an RR of 4.48 (95% *CI*: 3.46–5.81). Figure 3D displayed the mortality cohort effect for drowning, which also initially decreased and then increased from the 1927 cohort onward, with the highest cohort effect seen for the 1927–1931 cohort, exhibiting an RR of 2.33 (95% *CI*: 1.86–2.92).

## 4 Discussion

To the best of our knowledge, this was the first published study to explore the effects of the APC model on four leading injuries in Jiangsu, China. There was a downward trend in injury-related deaths except for unintentional falls over the period 2012–2021, which was consistent with the trend in a study of the national monitoring level over the period 2010–2019 (21). The study period experienced economic and structural developments accompanied by consequential social changes. These advances included urbanization, demographic changes, internal migration, education pursuits, poverty reduction efforts, health insurance implementation, and establishing related regulations and legislation (22). Changes in those factors may have contributed to an enhanced awareness of injury prevention (11), reduced exposure to hazardous situations (16), and prompt provision of assistance (22), hence resulting in the decline of injury-related mortality in Jiangsu Province.

The APC model showed that each of the factor of age, period and cohort had a specific association with injury-related deaths. Death risk increased significantly with age, and the risk of all four leading injuries was high in older people aged ≥60 years. On the one hand, older adult individuals’ hearing, eyesight, activity coordination, balance, risk judgment, and ability to handle emergencies were reduced, making them prone to injury, and the degree of injury was more serious (23). On the other hand, older adult people living alone, who lacked care from their children and performed more unhealthy behaviors than did married older adult individuals (24). In 2020, the average life expectancy of the Jiangsu population was 79.3 years (25), which was higher than the national life expectancy (77.3 years) (26). Thus, the aging population of Jiangsu province bears a heavy burden. The age effect on traffic accident mortality in this study is similar to that in India and South Korea (13, 27). Older adult people not only have poor physiological reserves but also limited ability to adapt to trauma. When there is a delay in trauma care after a road traffic accident, the risk of adverse outcomes for the older adult may be higher, hence the mortality rate is relatively higher (28). Pedestrians constitute the primary demographic among road users succumbing to traffic accidents in Jiangsu Province, comprising approximately 47.33% of fatalities resulting from such incidents. Both this study and the national results show that the mortality effect of falls for the older adult increases rapidly with age after 60 years old, which may be related to the gradual decline in physiological functions of the older adult, including weakened of muscle strength, decreased balance ability, and reduced sensory organ function (14, 29). According to our study, suicide was an important type of injury in the 20–60-year-old age group and the risk of death from suicide increased with age, especially for men. Studies on gender differences in suicide have shown that men had stronger suicidal intent than women and that they used lethal means, even when the same methods were used, resulting in a higher lethality rate than women (30–32). The increased susceptibility to suicide among the older adult may stem from diminished social connections post-retirement, limited economic resources, and exposure to both internal stressors (such as illness or disability) and external stressors (such as verbal abuse), rendering them more vulnerable to suicidal tendencies (33, 34). Consistent with the results of the Canadian study, older people in Jiangsu have replaced younger people as having the highest drowning mortality rate of any age group (35). Factors contributing to the risk of fall-related drowning fatalities among older individuals encompass pre-existing medical conditions affecting both physical and cognitive capacities, reduced mobility, heightened frailty, and specific environmental hazards (36). Older adult individuals are more likely to drown in environments such as bathtubs and pools due to their weakened physical functions (35). Meanwhile, studies have shown that drowning was a common form of suicide among older individuals (37).

The utilization of the joinpoint regression model allowed us to effectively capture the changing trends. The 2014 showed a significant turning point in the age-adjusted road traffic accident mortality rates among males, likely due to the second revision of the Road Traffic Safety Law that made drunk driving a criminal offense (38). A previous study has shown that the most effective interventions in preventing road traffic death was drink-driving enforcement, credited with saving over 60,000 lives annually (37). The overall suicide mortality rate in Jiangsu Province was on a downward trend, which could be attributable to the implementation of a series of government policies. In 2002, the Chinese government issued a directive to cease the production of certain highly toxic pesticides (39). Subsequently, the Regulations of the People’s Republic of China on the Administration of Pesticides implemented in 2008 established regulations governing the production, importation, transportation, storage, and sale of pesticides. Fatalities from road traffic injuries decreased between 2020 and 2021, presumably attributable to diminished exposure resulting from pandemic control measures (40). Additionally, there was a marginal decrease in suicide rates, plausibly linked to heightened mental health support during the pandemic (41). However, the incidence of drowning fatalities increased in 2020, consistent with patterns observed in Australia and the United States (42, 43), potentially due to disruptions in swimming and water safety education caused by lockdown measures, leaving a considerable portion of the population, particularly children, inadequately prepared for safe aquatic interactions (42).

This study found inverted V-shaped curves between cohort effects and injuries, with the highest risk of road traffic accidents observed in the birth cohort population from 1957 to 1961, and unintentional falls were observed in the birth cohort population from 1952 to 1956, consistent with previous studies (13, 14). This group was also the most active in social mobility and industrialization endeavors. China has experienced rapid development in the past few decades, with the number of motor vehicles increasing from approximately 1.7 million in 1980 to 11.5 million in 1996 (9, 44). Additionally, environmental pollution is a risk factor for fall-related injuries (14). There was evidence of an association between environmental pollutants and death from cardiovascular disease, cerebrovascular disease, lung cancer, and pneumonia (45). These diseases are strongly associated with the risk of fall-related injuries (46). The declining trend of the cohort RRs of mortality due to the four leading injuries for both sexes in the younger birth cohort was likely related to better education and urbanization. Previous studies have supported the association between lower educational attainment and a higher risk of injury mortality due to increased exposure and unsafe behavior (16, 47). People with better educational backgrounds often take more protective measures, such as safety training or learning personal rescue skills. With the development of urbanization and the improvement of infrastructure, people can receive timely treatment after injuries occur, and the chance of exposure to dangerous water, toxic substances, and other dangerous factors is reduced, so the mortality rates from various injuries decrease.

There are several limitations of this study. First, the study is primarily limited by the quality of the Jiangsu provincial population death registration system. Despite many efforts to validate the quality of the data and the widespread use of the dataset, the data may still be subject to underreporting and misclassification in reporting practices, making it possible that the completeness and accuracy of mortality data may be biased to some extent. Second, the period was just 10 years, which means that crossover was possible in assessing age, period, and cohort effects, and the distinction between age and period effects may not be clear (48). Third, similar to other APC studies, the influence of ecological fallacy was inevitable since interpreting results from population levels does not necessarily apply to individuals (47).

## 5 Conclusion

Although injury-related mortality in Jiangsu Province has decreased in the past 10 years, it is still relatively high. Injuries remain an important public health problem, seriously threatening the lives of residents in Jiangsu Province. Injury mortality varies with injury type, gender, age, and urban–rural distribution. The trend of injury mortality in Jiangsu Province has significant age, period, and cohort effects. With the arrival of an aging society, fall mortality in Jiangsu Province rose. Meanwhile, the older adult were also the focus of other injuries, and the risk of death increased with age. The improvement of mortality benefits from the improvement of education level, urbanization development, and the promulgation and implementation of laws and policies. Injuries can be prevented and controlled. We should take targeted measures to reduce the disease burden of injuries.

## Data availability statement

The original contributions presented in the study are included in the article material, further inquiries can be directed to the corresponding author.

## Author contributions

WD: Methodology, Writing – original draft, Formal analysis. RW: Methodology, Writing – original draft. XF: Writing – review & editing, Methodology. XW: Writing – review & editing. JY: Writing – review & editing. JZ: Supervision, Writing – review & editing. HY: Data curation, Methodology, Writing – review & editing.
